# Extensive tissue-specific expression variation and novel regulators underlying circadian behavior

**DOI:** 10.1126/sciadv.abc3781

**Published:** 2021-01-29

**Authors:** Maria Litovchenko, Antonio C. A. Meireles-Filho, Michael V. Frochaux, Roel P. J. Bevers, Alessio Prunotto, Ane Martin Anduaga, Brian Hollis, Vincent Gardeux, Virginie S. Braman, Julie M. C. Russeil, Sebastian Kadener, Matteo dal Peraro, Bart Deplancke

**Affiliations:** 1 School of Life Sciences, École Polytechnique Fédérale de Lausanne, Lausanne, Vaud 1015, Switzerland.; 2 Swiss Institute of Bioinformatics, Lausanne, Vaud, Switzerland.; 3 Department of Biology, Brandeis University, Waltham, MA 02453, USA.

## Abstract

Natural genetic variation affects circadian rhythms across the evolutionary tree, but the underlying molecular mechanisms are poorly understood. We investigated population-level, molecular circadian clock variation by generating >700 tissue-specific transcriptomes of *Drosophila melanogaster* (*w^1118^*) and 141 *Drosophila* Genetic Reference Panel (DGRP) lines. This comprehensive circadian gene expression atlas contains >1700 cycling genes including previously unknown central circadian clock components and tissue-specific regulators. Furthermore, >30% of DGRP lines exhibited aberrant circadian gene expression, revealing abundant genetic variation–mediated, intertissue circadian expression desynchrony. Genetic analysis of one line with the strongest deviating circadian expression uncovered a novel *cry* mutation that, as shown by protein structural modeling and brain immunohistochemistry, disrupts the light-driven flavin adenine dinucleotide cofactor photoreduction, providing in vivo support for the importance of this conserved photoentrainment mechanism. Together, our study revealed pervasive tissue-specific circadian expression variation with genetic variants acting upon tissue-specific regulatory networks to generate local gene expression oscillations.

## Teaser

A dataset of >700 tissue-specific transcriptomes of *D. melanogaster* reveals population-level molecular circadian clock variation.

## INTRODUCTION

Earth’s rotation results in daily cycles of light intensity, temperature, and atmospheric pressure. These oscillations are reflected in the biosphere as circadian rhythms, which are manifested in numerous species across the evolutionary tree (*1*). In multicellular organisms, the periodic patterns of daily oscillation are established at all levels. Intricately coordinated feedback loops of gene expression through rhythmic transcript production across multiple tissues (*2*, *3*) thereby produce complex behaviors such as cyclic locomotor activity and sleep-wake cycles (*1*). Given its universal presence, the circadian clock was found to affect various crucial organismal processes, such as the cell cycle (*4*), metabolism including drug-metabolizing enzymes (*5*, *6*), cognitive abilities (*7*), immunity (*8*), stem cells (*9*), and aging (*10*).

Despite its ubiquity, the circadian clock is not immune to genetic variation. For example, it is well established that genetic background affects the preference of activity time [larks and night owls (*11*)] or can even induce certain circadian clock-related pathologies, such as delayed sleep phase disorder (*12*). However, the underlying genetic and molecular mechanisms are still poorly understood. This is because natural circadian rhythm observations in humans are obstructed by differences in individual lifestyle and by the absence of standardized conditions during the monitoring phase. Model organisms are therefore well positioned to contribute to a better understanding of the genetics of circadian rhythms. For example, in the fruit fly, *Drosophila melanogaster*, more than half (68%) of the protein-coding genes are evolutionarily conserved in humans, including functional orthologs of core circadian regulators (*13*). Moreover, the fruit fly is advantageous over other model organisms by scalability, fast generation time, and the ability to perfectly define experimental conditions. Furthermore, the *Drosophila* circadian field has been a pioneer in the isolation and characterization of genetic variants to uncover the molecular basis of behavior. But that work was mainly based on loss-of-function mutants that display aberrant circadian behavior, and little is known on the role of natural genetic variation on circadian rhythmicity. The fly community benefits from access to the *Drosophila* Genetic Reference Panel (DGRP), which consists of over 200 genetically diverse fly lines that constitute a highly valuable resource of natural genetic variation in *Drosophila* (*14*, *15*). The DGRP has already been successfully used to identify genotype to phenotype relationships through genome-wide association studies for various traits (*16*–*18*). Recently, Harbison *et al*. (*16*) revealed extensive variation in period length and rhythmicity index among DGRP flies, two common circadian rhythm readouts. Moreover, several hundred putative causal variants were identified, clearly demonstrating the contribution of genetic variation to circadian phenotypic diversity.

To understand how these genetic variants affect circadian rhythms, we need to uncover the underlying regulatory mechanisms. However, there are still several major aspects of molecular circadian biology that are yet to be fully elucidated. These include (i) how the circadian clock controls tissue- or cell type–specific expression rhythms, which affects a wide variety of metabolic, physiological, and behavioral processes (*19*, *20*); and (ii) how such molecular rhythms vary among genotypes and, in doing so, affect circadian behavior along these same processes. To address these key questions, we conducted a systematic analysis of circadian gene expression in the reference *w^1118^* line across four major tissues: brain, gut, Malpighian tubules, and fat body via temporal profiling of these tissues for two consecutive days resulting in 233 transcriptomes. This is, to our knowledge, the largest circadian dataset produced in flies to date and allowed us to identify >1700 mostly tissue-specific circadian (TSC) genes, substantially expanding the catalog of known clock-controlled genes. Analysis of the underlying gene regulatory network (GRN) based on cycling genes revealed transcription factors (TFs) that may control tissue-specific, circadian gene expression. In addition, we uncovered seven previously uncharacterized circadian regulators, which we validated by perturbation assays. We also assessed the influence of genetic variation on circadian rhythm by performing high temporal resolution RNA sequencing (RNA-seq) on 141 DGRP lines across three tissues, yielding another 451 transcriptomes. This screen revealed that 45 (>30%) of the sampled DGRP lines exhibit aberrant circadian gene expression. Since this variation is mostly manifested in a tissue-specific manner, our findings reveal extensive molecular circadian desynchrony between tissues. To understand the underlying molecular mechanisms, we performed a genetic analysis of the line (DGRP-796) showing the strongest deviating circadian expression. Through genetic and molecular analyses, protein structural modeling and simulation, as well as brain immunohistochemistry, we found that this line features a dysfunctional clock in both brain and peripheral tissues driven by a novel *cry* mutation that disrupts the light-driven flavin adenine dinucleotide (FAD) cofactor photoreduction. Hence, we validated in vivo the importance of this evolutionary conserved photoentrainment mechanism in the circadian pacemaker. Together, these results underscore the resource value of the generated tissue- and genotype-specific gene expression atlas in informing on previously unknown circadian biology and the effect of genetic variation thereon.

## RESULTS

### Tissue-specific cycling genes and putative core circadian components

**Fig. 1:**
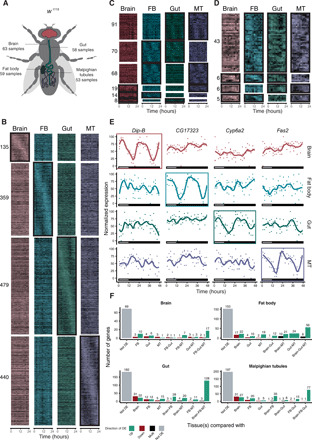
Tissue-specific gene expression cycling and detection of previously uncharacterized circadian regulators based on the reference *w^1118^* time series experiments. (**A**) Schematic representation of the tissue-specific samples obtained from *w^1118^*. (**B**) Heatmap of the expression of TSC genes averaged across 2 days of observation. The presence of the characteristic circadian patterns in the tissue is indicated with black rectangles around the heatmaps. The number at the left designates the number of detected TSC genes. (**C** and **D**) Heatmap of cycling in two (C) and three tissues (D) gene expression averaged across 2 days of observation. Black rectangles indicate tissues in which genes were found to be cycling. For (B) to (D), lighter areas or the heatmaps correspond to lower expression levels. The time is indicated at the bottom of the heatmap. (**E**) Examples of expression across tissues of the TSC genes during two consecutive days of observation (12-hour/12-hour LD + 24-hour DD). The rectangle at the bottom shows the presence and absence of light. (**F**) The majority of TSC genes are not tissue-specifically expressed. Each panel is dedicated to the genes found cycling only in the corresponding tissue. The *X* axis denotes the expression pattern of genes across tissues, and the *Y* axis shows the number of TSC genes with the considered expression pattern. The color of the bar indicates a differential expression status of the gene compared to specific tissues as listed on the *X* axis. Black (“Multi”) indicates genes with a varying direction of differential expression. In (B) to (E), FB stands for fat body and MT for Malpighian tubules, respectively. DE, differentially expressed.

To investigate TSC gene expression, we first created a baseline gene expression time series using the *w^1118^ D. melanogaster* strain. We sampled the brain, gut, Malpighian tubules, and fat body of this reference strain (Fig. 1A and table S1) every 2 hours, over 48 hours, in triplicates. For this, we used one 12-hour/12-hour light-dark (LD) cycle followed by 24 hours in darkness (DD) at 25°C to filter genes that were merely induced by light. Thereafter, we generated genome-wide expression profiles using a high-throughput, 3′end counting RNA-seq method, bulk RNA barcoding (BRB-seq) (*21*). We used JTK CYCLE (*22*) to determine gene expression periodicity and rhythmicity and detected 1757 cycling genes across four tissues (Fig. 1, B to D, and table S2) with most of these cycling in a tissue-specific manner (Fig. 1, B and E). This finding is consistent with the observation that clock output rhythms are generated locally (*2*, *3*), although the absolute majority of the TSC genes were found to be expressed in more than one tissue [brain: 95.6% (129 of 135), fat body: 98.3% (353 of 359), gut: 96.0% (460 of 479), Malpighian tubules: 98.4% (433 of 440)]. In particular, 38 to 51% of TSC genes did not exhibit a change in the expression level compared to any other tissue (Fig. 1F). Nonetheless, a notable proportion of TSC genes was up-regulated in the tissue where we found them to cycle compared to the other three tissues. Similar to the whole set of TSC genes, the majority of genes that were up-regulated in the tissue where they also cycled were still expressed in more than one tissue (brain: 12 of 17, fat body: 52 of 56, gut: 109 of 128, and Malpighian tubules: 70 of 77). These findings suggest that the mechanism by which tissue-specific cycling is achieved tends to be decoupled from the mechanism that drives tissue-specific expression, although sometimes they can go hand in hand. Not differentially expressed TSC genes were enriched in gene ontology (GO) terms related to intracellular transport in all four tissues [brain: GO:0046907, intracellular transport, adjusted *P* value (*P*_adj_) = 0.028; fat body: GO:1902533, positive regulation of intracellular signaling, *P*_adj_ = 0.019; gut: GO:0006886, intracellular protein transport, *P*_adj_ = 0.003; and Malpighian tubules: GO:0046907, intracellular transport, *P*_adj_ = 0.003], while GO analysis of the TSC genes that were up-regulated in the tissue where they also cycled did not reveal any significantly enriched terms.

**Fig. 2:**
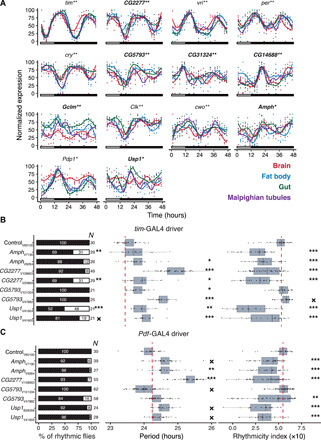
Genes cycling in all examined tissues. (**A**) Rhythmic expression of the canonical circadian genes and previously uncharacterized genes (bold) cycling in all four tissues during two consecutive days (12-hour/12-hour LD + 24-hour DD). Rectangles show the presence and absence of light. (**B** and **C**) Knockdown effect of four putative core circadian genes in *tim*-expressing (B) and *Pdf*-expressing (C) clock neurons (LN_v_s) on locomotor behavior: % rhythmic flies (left), period (middle), and rhythmicity index (right). The *Y* axis indicates gene names, and the subscript denotes an RNAi line stock identifier. The first letter of the subscript designates the RNAi line stock center: (B)loomington and (V)DRC. Column *N* specifies the number of flies used in the experiment. The red dashed line depicts the mean of measurements in control flies. For the period plot, only measurements on rhythmic flies were used. A test of equal proportions was used to assess the statistical significance of the % rhythmic flies deviating from the control, and analysis of variance (ANOVA) was used for period and rhythmicity strength. Several RNAi lines were tested per gene. *Amph*, *CG2277*, *CG5793*, and *Usp1* showed robust and consistent effects in both RNAi constructs and both gene drivers. Figure S1 displays an overview of all tested lines. The stars indicate the FDR-adjusted *P* values: ***P < 0.001, **P < 0.01, *P < 0.05, and ✕*P* < 0.1.

Our transcriptomic analyses also revealed that only 14 genes cycled in all tissues. These included all main clock genes: *tim*, *vri*, *per*, *cry*, *Clk*, *cwo*, and *Pdp1*, benchmarking our approach, as well as seven largely uncharacterized ones: *CG2277*, *CG5793*, *CG31324*, *CG14688*, *Gclm*, *Amph*, and *Usp1* (Fig. 2A). To determine whether the latter genes play a role in the circadian pacemaker system, we explored whether their knockdown in all clock cells using the *tim*-GAL4 driver, or in the *Pdf*-expressing central clock neurons [ventrolateral neurons (LN_v_s), using *Pdf*-GAL4], would cause locomotor activity rhythm defects, the most common readout for circadian rhythm integrity. To provide a robust evaluation, two upstream activating sequence–RNA interference (UAS-RNAi) constructs targeting different gene regions were used. Overall, knockdown of all tested genes, except *CG14688* for which only one RNAi line was tested, affected the locomotor behavior either by affecting the number of rhythmic flies or by altering their period, rhythmicity strength, or rhythmicity index (a readout of the strength of the periodic pattern) with at least one pair of UAS-RNAi lines using either the *tim* or *Pdf* drivers (fig. S1, and tables S3 and S4). Yet, locomotor activity levels were not decreased (fig. S1, A and B). Four genes: *Amph*, *CG2277*, *CG5793*, and *Usp1* showed robust and consistent effects in both RNAi constructs and both gene drivers, i.e., *tim* and *Pdf* (Fig. 2, B and C). Specifically, we found that, upon knockdown of *Amph*, *CG2277*, and *Usp1*, the oscillation period increased and the rhythmicity index decreased. In contrast, *CG5793* knockdown resulted in an increased period for the *tim*-GAL4 driver and a decreased one for *Pdf*-GAL4, while the rhythmicity index increased under the tim-GAL4 driver and decreased under *Pdf*-Gal4 (Fig. 2, B and C). However, the knockdown effect size for period length and rhythmicity index was only mild, i.e., the period varied between 23.8 and 25.1 hours compared to the period shortening to 16 and 20 hours or its elongation up to 32 hours in case of actual core clock gene disruption (*23*, *24*). Nonetheless, *Usp1* knockdown did significantly reduce the percentage of rhythmic flies in all RNAi constructs using the *tim*-Gal4 driver.

**Fig. 3:**
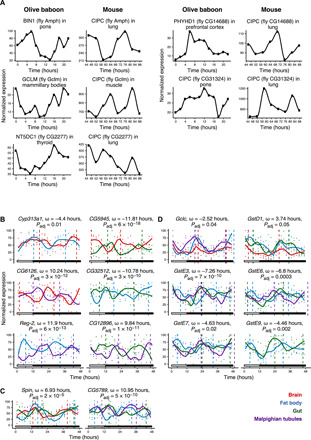
Mammalian orthologs of genes cycling in all tissues and phase shift in the expression across tissues. (**A**) Putative core clock regulators orthologs’ expression in baboon and mouse. The data were derived from (*27*) and (*26*), respectively. (**B**) Examples of genes cycling in two tissues with the most significant phase shift. ⍵ in plot headers denotes the phase shift value. Vertical lines correspond to the tissue-specific expression’s molecular peak times. Tissue-specific molecular peak times on the second day were calculated as the sum of tissue-specific molecular peak time on the first day and tissue-specific gene period. (**C**) Examples of genes cycling in three tissues with the most significant phase shifts per tissue combination. *Spin* and *CG5789* have a phase shift in all pairs of tissues they cycle in. ⍵ in the plot header for *Spin* denotes the phase shift between the brain and gut. Phase shifts for the brain–fat body and fat body–gut were 3.72 and 3.20 hours, respectively. ⍵ in the plot header for *CG5789* denotes the phase shift between fat body and gut. Phase shifts for the fat body–Malpighian tubules and gut–Malpighian tubules were 5.50 and −5.45 hours, respectively. (**D**) Phase shift in the expression patterns of glutathione metabolism–associated genes.

Since the circadian clock is conserved across species (*25*), we sought to investigate whether these seven largely uncharacterized genes have functional homologs that might play a role in the mammalian clock pacemaker. We found that five of them (*Amph*, *Gclm*, *CG14688*, *CG31324*, and *CG2277*) have orthologs in mouse (*Mus musculus*), baboon (*Papio anubis*), and human (*Homo sapiens*) (table S5). By assessing circadian gene expression data across tissues in mouse and baboon from databases and previous publications (*26*, *27*), we found that all of them cycled across multiple tissues in baboon, and *Amph*, *Gclm*, and *CG14688* orthologs cycled in at least one tissue in the mouse (Fig. 3A). These observations, together with our own RNAi results, support the notion that the genes that cycled across tissues in *Drosophila* might be as yet uncharacterized but conserved members of the molecular circadian clock machinery or central elements in the transmission of circadian information to other biological processes.

While most cycling genes were unique to one tissue and only 14 genes cycled in all four tissues, as described above, 344 (19.6%) cycled in two or more tissues (Fig. 1, C and D), providing an opportunity to study circadian expression desynchrony between tissues. This is relevant given that the alignment of clocks throughout an organism is the key for proper circadian functioning (*28*) and that certain misalignments may lead to various pathologies. However, the extent of molecular circadian synchrony across tissues is still poorly understood. To address this, we used CircaCompare (*29*) to perform an intertissue phase shift analysis for the genes that cycled in two or more tissues. This revealed 101 genes [29%, false discovery rate (FDR) < 0.05] that display a phase shift in expression of >2 hours between at least one pair of tissues in which they cycle (Fig. 3, B to D, and table S6). The threshold on the phase shift value was dictated by the sampling frequency of the experiment. GO enrichment analysis of the phase-shifted genes showed enrichment in both “glutathione metabolic process” (biological process; Fisher’s exact test, *P* = 5.1 × 10^−5^) and “glutathione transferase activity” (molecular function; Fisher’s exact test, *P* = 2.2 × 10^−3^). Analysis of the expression profiles of glutathione-associated genes (*Gclc*, *GstD1*, *GstE3*, *GstE6*, *GstE7*, and *GstE9*) indeed showed a systematic molecular desynchronization across tissues (Fig. 3D). Specifically, while the expression profiles of these genes were synchronized between the gut and Malpighian tubules, they were significantly delayed in the fat body with phase shift values ranging from 4.5 to 7.8 hours (Fig. 3D). Only two genes (*GstD1* and *Gclc*) in this category were cycling in both brain and peripheral tissues (fat body and gut for *Gclc*; fat body and Malpighian tubules for *GstD1*) (Fig. 3D). Furthermore, *GstD1* and *Gclc* were found to be targeted by Clk (table S2) (*2*), suggesting that this core circadian regulator directly affects their temporal expression profiles. Together, these findings suggest that glutathione metabolism is not just under circadian control, with *Gclc*, *Gclm*, and *GstD1* contributing to the early circadian regulatory cascade in agreement with previous studies (*30*, *31*), but that this control is realized in a tissue-specific, desynchronized manner. To our knowledge, this is the first report of a naturally occurring tissue-specific, circadian molecular desynchrony of a biological process in *D. melanogaster*.

### A regulatory TF hierarchy drives tissue-specific, circadian gene expression

**Fig. 4:**
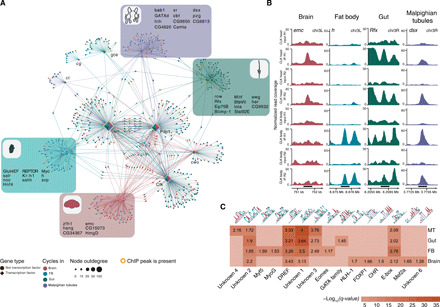
Regulatory network and motif analyses of TSC gene expression. (**A**) GRN based on genes cycling in at least one tissue. The color of the node denotes the tissue(s) a gene cycles in. The size of the node designates the node out-degree. The color of the edges represents the tissue in which the edge was derived. Shaded areas indicate the TSC gene regulatory modules with the names of the TFs arranged in the same order as the nodes. TFs are denoted as diamonds. Nodes with an orange rim indicate direct targets of Clk/Cyc. For network clarity, only the top 200 links ranked by weight from every tissue are visualized. (**B**) Examples of Clk/Cyc binding profiles at the vicinity of TSC TF–coding genes as derived from (*2*). The name of the TSC TF–coding gene is indicated at the top left corner of each column. Rows correspond to ChIP-seq samples, and columns denote tissues for which a cycling TF-coding gene was found to be specific. The black bar at the bottom of each column designates the ChIP-seq peak location. (**C**) Motif enrichment in TSC gene promoters. The motif’s name is indicated at the bottom, and its graphical representation is depicted at the top. The number inside cells refers to the fold enrichment of a motif in the target regions over the random background. The color bar indicates the *q*-value for motif detection.

To uncover global principles and major functional modules of the TSC pacemaker, we built a GRN based on all detected cycling genes and visualized the top 200 most significant nodes with Cytoscape (Fig. 4A) (*32*, *33*). Given the key role of genes such as *Clk*, *cwo*, *vri*, and *Pdp1* in clock control in all tissues, we expected these genes to be heavily interconnected, located centrally in the network and bridging different nodes in all four tissues. Fifty-two percent of TSC genes were directly connected to these regulators (Fig. 4A). To decipher direct targets of core clock circadian regulators such as Clk and putative genes located downstream of a regulatory cascade, we incorporated information about Clk binding sites from publicly available chromatin immunoprecipitation sequencing (ChIP-seq) data (*2*). This revealed that 20% of TSC genes were directly bound by Clk/Cyc, indicating that many clock targets are directly activated by clock master regulators (Fig. 4, A and B) (*2*, *3*). This master clock GRN was also connected to several smaller modules that were composed of tissue-specific, cycling TFs. This observation suggests a second level of circadian regulation in the clock hierarchy in which tissue-specific TFs that are not part of the master pacemaker act as integrators of circadian information to generate local gene expression rhythms (*2*, *3*). Most of the tissue-specific cycling TFs (37 of 38) showed tissue-specific cycling but not tissue-specific expression, pointing to further unexpected regulatory complexity in local gene expression rhythmicity.

Compelling evidence suggests that context-specific TF binding is achieved by partner TFs that contribute to the identification of binding targets (*34*). To identify other TFs that might play a role in TSC expression but are not necessarily cycling, we performed a motif enrichment analysis around the transcription start site of TSC genes (Fig. 4C). As expected, we observed that all four tissues are enriched for E-box sites, which are known to be bound by Clk/Cyc in upstream activating sequences in all tissues (*2*, *35*). In addition, we detected several other motifs such as *Drosophila* homolog DNA replication‑related element‑binding factor (DREF) and the so far uncharacterized unknown motifs 1 and 2 to be enriched in all tissues (Fig. 4C), suggesting that the clock master GRN might involve other regulators that are yet to be identified. We also found several motifs that might mediate TSC gene expression rhythms such as GATA in the gut (*36*) and Mef2a in the brain, whose knockdown leads to the abolishment of circadian behavior (*37*, *38*). Moreover, we discovered that the binding motif for Foxo is enriched in the brain, and since *foxo*-deficient flies are arrhythmic when exposed to oxidative stress (*39*), this finding supports a role for Foxo in the fly brain clock. Together, our analyses provide a comprehensive overview of the regulatory subnetworks underlying ubiquitous and TSC gene expression. These networks might be further expanded by (i) uncovering TFs associated with uncharacterized motifs and (ii) determining the binding specificity of yet-uncharacterized tissue-specific TFs, including *emc*, *CG34367*, and *HmgD*—the most prominent brain-specific hub components.

### Large-scale genetic variation affects circadian rhythm

**Fig. 5:**
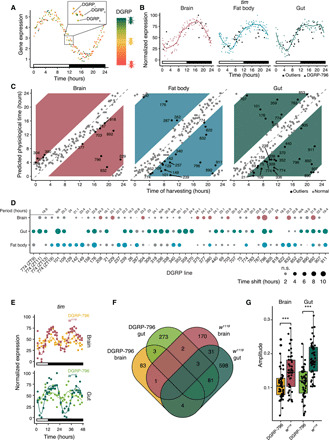
Transcriptomic-based physiological circadian time assessment for tissue-specific DGRP profiles. (**A**) Experimental strategy to reconstruct dynamic circadian expression profiles based on static transcriptomes from DGRP lines. Dots represent the expression value of the same circadian gene derived from the transcriptome of a random DGRP line sampled at the time indicated on the *X* axis. (**B**) The dots recapitulate the dynamic expression pattern of a cycling gene as it would be derived from a regular time series analysis (shown for timeless). (**C**) MTT-inferred physiological time versus sampling time. Colored areas feature a difference of >3.4 hours between the physiological and sampling times. (**D**) Time shift of outlier lines across tissues. Gray circles indicate a nonsignificant (n.s.) shift <3.4 hours, and the absence of a circle denotes missing data. The period value was derived from (*16*). The first four columns show samples for line DGRP-774 used as a proof of concept (see Materials and Methods), which has an 18.9-hour period in males (*16*). N/A, not applicable. (**E**) Brain and gut expression profiles of timeless during a 12-hour/12-hour LD followed by 24-hour DD in *w^1118^* and DGRP-796. The rectangle indicates light presence versus absence. (**F**) Venn diagram showing the overlap between cycling genes in *w^1118^* and DGRP-796 time series in brain and gut. The color code is as in (E). (**G**) Amplitude distribution of the top 50 cycling genes ranked by the amplitude detected in the *w^1118^* and DGRP-796 time series. ***P < 0.001; *t* test (*N* = 50).

Having established a reference atlas of cross-tissue circadian transcriptomes allowed us to investigate the impact of genetic variation on TSC gene expression using the DGRP. To alleviate the tremendous challenge of having to profile each of 141 available DGRP lines every 2 hours for two consecutive days across several tissues, we implemented an alternate approach that was inspired by the concept of reconstructing dynamic profiles from static samples (*40*). To do so, we collected each of the 141 DGRP lines only once. Yet, the sampling of the DGRP collection was done at a very high frequency (~9-min interval between each line; Fig. 5A). Then, we performed BRB-seq on the dissected brains, guts, and fat bodies. In total, we acquired 338 static transcriptomes (105, 129, and 104 for the brain, fat body, and gut, respectively) at high temporal frequency. In addition, three DGRP lines that displayed a normal locomotor circadian rhythm (*16*): DGRP-208, DGRP-321, and DGRP-536 were sampled around the clock every 4 hours for 24 hours in case of DGRP-208 and every 2 hours for 24 hours otherwise, resulting into 30, 29, and 25 samples for the brain, fat body, and gut, respectively (table S1). As indicated, these sampled DGRP lines were selected on the basis of their regular circadian rhythm, thus allowing us to estimate natural variation in circadian gene expression levels in lines that do not exhibit irregular behavioral circadian rhythms. Notably, most of the sampled DGRP lines for which period measurements were available [81 (65%)] featured a circadian period length close to normal, ranging from 22 to 26 hours, whereas no data were available for 15 lines (table S1) (*16*). The analysis of temporal core clock gene profiles that were formed by integrating individual DGRP samples revealed clear cycling profiles for several known circadian genes (Fig. 5B and fig. S2A). These results demonstrate that we were able to reconstruct dynamic cycling patterns from statically collected samples.

Subsequently, to investigate the impact of genetic variation on circadian gene expression, we computed the physiological circadian time of each static DGRP sample. In essence, we determined how much the static transcriptome of each line deviates from the expected transcriptome at the point of day at which the line was collected. To calculate the physiological time, we assessed several published computational methods next to two in-house developed ones described below. In general, for every evaluated technique, published or newly developed, we divided the process of physiological time inference based on static transcriptomes into two stages. In a first step, we created a reference time series of gene expression followed by a statistical inference of the relationship between a multidimensional gene expression matrix and time, i.e., selection of the best predictor genes. To create a reference time line, we used the previously introduced *w^1118^* dataset combined with the three profiled DGRP lines (DGRP-208, DGRP-321, and DGRP-536). The high temporal sampling frequency of the *w^1118^* dataset as well biological replication allowed us to account for technical variability in the expression values. Furthermore, by also considering the three profiled DGRP lines, we were able to include in the model benign expression variation that does not significantly alter the circadian clock, i.e., that does not cause circadian phenotypic alterations. In a second step, we mapped a gene expression vector of a static DGRP sample against the reference time line that was created in the first step. In other words, we ranked in time each DGRP line based on its gene expression profile relative to the reference, allowing us to compute a physiological time per line. This strategy allowed us to infer putative alterations of the molecular circadian clock based solely on single static transcriptomes without performing the challenging temporal profiling of each DGRP line.

As indicated above, we compared several computational approaches for inferring the physiological time: an in-house developed least absolute shrinkage and selection operator (LASSO) and directional statistics (DirectStat) method, a neural network, as well as the molecular time table (MTT) (*41*) and ZeitZeiger (*42*) strategies (fig. S2B). Because of the limited number of samples, we used the “leave-one-out” approach, a widely established machine learning technique, to assess the performance of each algorithm. To prevent model overfitting on the static transcriptomes, only *w^1118^* and the three profiled DGRP lines (DGRP-208, DGRP-321, and DGRP-536) were used as a training set. During the evaluation, a sample was taken out of the training set, and an algorithm was trained on the remaining samples, followed by the prediction of the physiological time of the removed sample. This procedure was repeated on all samples from the training set, and the difference between the predicted physiological time and time of harvesting was calculated. Since no changes in circadian rhythm were observed for either *w^1118^* or the three profiled DGRP lines, the difference between predicted time and harvesting time was used as the main metric for method evaluation. We found that the MTT approach yielded the lowest averaged difference between predicted and harvesting times across the training set (1.32 hours). We therefore considered this approach to be the most reliable (fig. S2B), reaching a precision, here conservatively defined as three SDs from the mean, of 3.4 hours. This precision is in our opinion rather remarkable considering the 2-hour sampling frequency of our *w^1118^* experiment.

As a proof of concept that phenotypically relevant deviations in molecular rhythms could be detected on the basis of static transcriptomes, we acquired several samples (11 for brain, 11 for fat body, and 7 for gut) at various time points for the DGRP-774 fly line, which has been shown to feature a shortened circadian period (20 hours) (*16*). While DGRP-774 does not display the biggest deviation from normality in terms of period length across the DGRP, we note that the efficiency of detecting molecular circadian rhythm deviations from static transcriptomes does not clearly correlate with the absolute value of the deviation (see Materials and Methods and fig. S2C). We found that one fat body and three gut samples of DGRP-774 (Fig. 5, B to D) have indeed a predicted physiological time that significantly (>3.4 hours) differs from the time of harvesting, providing support to the validity of our approach. Moreover, using JTK CYCLE, we estimated the molecular period based on *tim* expression pattern of this line to be 22 and 20 hours in the brain and fat body, respectively, which is in line with the measured behavioral period of 18.9 hours by Harbison *et al*. (*16*). However, as indicated, we identified no DGRP-774 brain samples with a predicted physiological time that could be significantly distinguished from that of the harvesting time, according to the model (Fig. 5, B to D). The root of this observation is unclear at this point but may be both biological and/or technical in nature (see Discussion).

Using the MTT strategy, we then inferred the physiological circadian time per tissue for every DGRP sample (Fig. 5, C and D, and table S7). We thereby note that the method was executed separately for every tissue to avoid imposing the conceptual restriction that all tissues should adhere to the same physiological time. This revealed a remarkable 45 DGRP lines (32.1%) with a physiological time >3.4 hours shifted from the harvesting time. Most of these lines exhibited molecular circadian variation only in one or two tissues (Fig. 5D), reemphasizing the observed synergy between the circadian clock and tissue-specific regulatory networks (Fig. 4A). Among lines showing aberrant physiological time, two of them, DGRP-796 and DGRP-892, had a significant out-phased or disrupted clock in all three examined tissues, suggesting that a main clock component is affected in these lines. Supporting our approach, DGRP-892 flies have recently been shown to have a period of 31 hours, yet the period for the other line was close to normal (*16*). To provide insights into the genetic mechanisms underlying the observed molecular circadian variation and to distinguish between the phase shift and disruption scenarios, we focused on DGRP-796, since it showed the most notable (>10 hours) out-phased molecular clock. We sampled the brains and guts from DGRP-796 flies every 2 hours for 48 hours (12-hour/12-hour LD followed by 24 hours DD at 25°C), performed BRB-seq, and compared the results to the *w*^1118^ reference (Fig. 5, E to G, and table S2). None of the core clock genes (except *Pdp1* in the gut) cycled with a circadian period (24 hours) in either the brain or gut in DGRP-796 (Fig. 5E and fig. S3A), and overall, we observed a marked reduction in the total number of cycling genes in DGRP-796 compared to *w*^1118^ (91 versus 207 in the brain and 362 versus 717 in the gut; Fig. 5F). Moreover, the amplitude of the top 50 cycling genes in DGRP-796 was significantly reduced compared to *w*^1118^ in both tissues (two-sided *t* test *P* = 0.00012 for the brain and *P* = 5 × 10^−11^ for the gut; Fig. 5G). Of the remaining genes that we found cycling in DGRP-796, only one was shared with *w*^1118^ in the brain and 84 in the gut. Together, these results strongly suggest that DGRP-796 suffers, in fact, from a nonfunctional molecular clock.

**Fig. 6:**
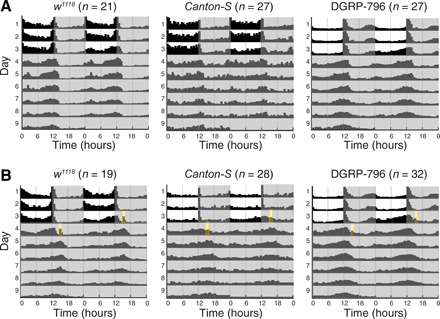
Behavioral characterization of DGRP-796. (**A**) Double-plotted activity measurements for *w^1118^* (right), *Canton-S* (middle), and DGRP-796 flies (left) in DD after entrainment in standard LD conditions. The number of flies used in each experiment is indicated in the parenthesis next to the genotype. Each row of the diagram represents a histogram of flies’ activity during the day concatenated with the data from the previous day. The first row displays the data for observation days 1 and 2, the second row shows day 2 and 3, etc. The dark gray background indicates the absence of light, and white background designates the presence of light. The activity of *w^1118^* focused in two peaks at dusk and dawn, and that of DGRP-796 was concentrated mostly around dusk, whereas *Canton-S* was active uniformly across the day. (**B**) Double-plotted activity measurement of *w^1118^* (right), *Canton-S* (middle), and DGRP-796 flies (left). The yellow rectangle indicates the light pulse of 20 min (CT15). Upon subjection to the light pulse, *w^1118^* and *Canton-S* showed a phase delay of approximately 3.4 hours, which could be seen as a shift to the bottom right of the activity peak between the diagram rows. DGRP-796 did not respond to the light pulse.

Given its severely dampened rhythms in gene expression, we next assessed whether DGRP-796 also has aberrant locomotor rhythmic behavior. Unexpectedly, DGRP-796 flies showed standard bimodal activity patterns under LD and rhythmic circadian activity patterns under DD, with a period of around 24 hours (Fig. 6A). This phenotype, i.e., the lack of molecular rhythms associated with normal locomotor activity rhythms in DD, points to a mutation in cryptochrome (*cry*). This gene was initially isolated in a screen for mutants with dampened rhythmic *per* gene expression (*43*, *44*). The *cry* gene codes for a blue-light photoreceptor involved in the *Drosophila* circadian pacemaker light input pathway. To test whether the light input pathway was indeed disrupted in DGRP-796 flies and affected behavioral patterns, we performed a light pulse (20 min, 600 lux) on DGRP-796 and control *w^1118^* and *Canton-S* flies in the subjective late evening (CT15), the time when the clock is most sensitive to light input (*45*). The *Canton-S* genotype was introduced to account for possible differences in light perception between *w^1118^* and DGRP-796 due to eye pigmentation. Our results showed that, while *w^1118^* and *Canton-S* flies have a phase delay of approximately 3.4 hours, DGRP-796 flies do not respond to the light pulse (Fig. 6B).

**Fig. 7:**
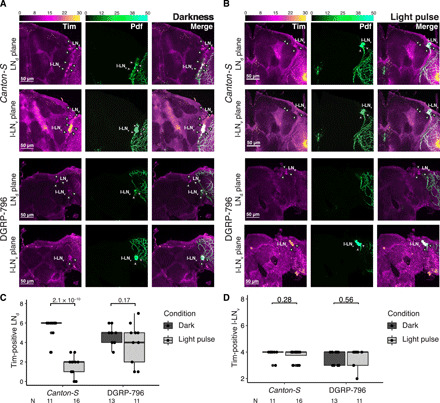
Histological characterization of DGRP-796. (**A**) In the absence of light, Tim (first column) is expressed in LN_d_s and l-LN_v_ neuronal groups in both *Canton-S* and DGRP-796. L-LN_v_s are also marked by expression of Pdf (second column). No significant difference is observed between the fly lines. (**B**) Influence of a light pulse on neuronal groups in *Canton-S* and DGRP-796. Upon light pulse exposure, Tim is effectively degraded in the LN_d_s in *Canton-S* (first row) but to a much lesser extent in DGRP-796 [third row versus no light pulse (B)]. Large LN_v_s expressing Pdf (second column) are not affected by the light pulse in either of the lines (second and fourth rows) (*47*), constituting an internal technical control. (**C**) Quantification of Tim staining in LN_d_s in *Canton-S* and DGRP-796. (**D**) Quantification of Tim staining in l-LN_v_s in *Canton-S* and DGRP-796. The number of Tim-expressing LN_d_s (C) and l-LN_v_s (D) before and after the light pulse was counted in both fly lines, and a two-sided *t* test was performed. The number of used brains is indicated at the bottom.

To subsequently elucidate whether the observed disruption of the light input pathway in DGRP-796 manifested itself also at a physiological level, we performed a brain immunohistochemistry assay. Specifically, since Tim is rapidly degraded after exposure to light (*46*), we reasoned that DGRP-796’s failure to respond to light should be visible in the form of reduced or even absent Tim degradation in the pacemaker neurons. To investigate this, we used α-Tim and α-Pdf antibodies to label all clock cells and large LN_v_s (l-LN_v_s), respectively. The number of l-LN_v_s as well as dorsal LN_v_s (LN_d_s) in reference lines (*w^1118^* and *Canton-S*) and DGRP-796 was assessed at ZT21 (peak expression of Tim) before and after a 30-min light pulse (Fig. 7, A to D, and fig. S3, B and C). We did not observe any changes in Tim or Pdf distribution across neuronal groups (l-LN_v_s and LN_d_s) between the fly lines before the light pulse (Fig. 7A and fig. S3B). However, after the 30-min light pulse, we noticed significantly reduced Tim degradation in the LN_d_s in DGRP-796 compared to the reference line (two-sided *t* test, *P* = 0.17 for DGRP-796, *P* = 2.1 × 10^−10^ for *Canton-S*, and *P* = 0.043 for *w^1118^*; Fig. 7, B and C, and fig. S3, B and C). As expected, we observed no significant difference in the number of Tim/Pdf-positive l-LN_v_s before and after light exposure, which constitutes an internal technical control (two-sided *t* test, *P* = 0.56 for DGRP-796, *P* = 0.28 for *Canton-S*, and *P* = 0.878 for *w^1118^*; Fig. 7, B and D, and fig. S3, B and C). While previous studies reported Tim photodegradation by Cry in l-LN_v_s (*47*, *48*), we note that the experimental conditions of these studies were different from ours. Flies were kept in DD for an extra hour in (*47*, *48*), while our measurements were performed directly after the light pulse, aiming to measure immediate effects. In view of the commonly accepted molecular model of the phase being reset by a light pulse in *Drosophila* (*43*, *44*, *49*), the observed decrease in Tim degradation in LN_d_s in DGRP-796 suggests a significantly affected or even disrupted light input pathway, likely mediated by Cry.

These findings prompted us to perform a sequence analysis of *cry* over the whole DGRP. We discovered that, in total, 322 variants affect *cry* across the panel. Of these, we found that only 21 are nonsynonymous, with 19 being single-nucleotide polymorphisms (SNPs) and the other two a frameshift ACGAAA > GG at position 3R 15040256 and a codon change plus codon deletion TGTGGGT > T at position 3R 15040481, while the rest fall in upstream, downstream, 3′ untranslated region or intronic regions, or are plain synonyms. We noticed that the frameshift is specific to the DGRP-356, while the in-frame deletion is also present in DGRP-355, DGRP-796, and DGRP-911. In this study, DGRP-355 and DGRP-356 were not sampled. Consequently, the only variant that affects *cry* that could also be causal to the phenotype was TGTGGGT > T. However, as this codon change plus codon deletion is in fact an in-frame deletion and as it does not affect a known functional region, we decided to also scan other known clock, clock-controlled and light input DGRP-796 genes for potentially disruptive mutations that could modulate the light input/response pathway. Yet, we did not uncover any obvious loss-of-function mutations or indels.

**Fig. 8:**
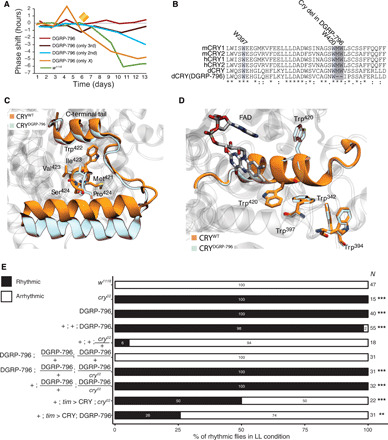
Effect of the identified novel cry allele on protein structure. (**A**) Chromosome mapping strategy involving phase shifts of *w^1118^*, DGRP-796, and their crosses after the light pulse at CT15 (yellow diamond). (**B**) Alignment of Cry protein sequences for the wild-type (WT) dCry, DGRP-796 (dCry DGRP-796), and its mouse (mCry1 and mCry2) and human (hCry1 and hCry2) homologs. (**C**) WT (yellow) and DGRP-796 (light blue) Cry protein structure models. (**D**) Enlarged *w^1118^* (yellow) and DGRP-796 (light blue) Cry protein structure models in the Asp^410^-Arg^430^ region. The DGRP-796 *cry* mutation disrupts the secondary structure of the ⍺ helix and causes a reorientation of Trp^420^. (**E**) The percentage of rhythmic and arrhythmic flies in *w^1118^* mutant flies under LL conditions. The numbers at the right indicate population size. FDR-adjusted *P* values: ***P < 0.001, **P < 0.01, *P < 0.05 and ×*P* < 0.1.

To identify the potential responsible gene(s) in DGRP-796, we therefore decided to use a chromosome mapping strategy using the lack of phase shift response as a readout (Fig. 8A). These experiments revealed that the individual swapping of both the second and third *w^1118^* chromosomes with the respective DGRP-796 chromosomes triggered a phenotypic effect (Fig. 8A). Specifically, chromosome 2 swapping led to a reduced phase response to a light pulse (with a value in between those of *w^1118^* and DGRP-796), suggesting that phase response-influencing variants may be located on chromosome 2. This prompted us to perform a variant scan of light- or circadian rhythm–associated genes on chromosome 2, revealing 87 nonsynonymous variants across 38 genes, 11 premature start gains in 10 genes, three codon deletions (in *Akap200*, *mus201*, and *Mef2*), and one codon change with codon insertion in *Akap200*. Yet, given that DGRP-796 is (as far as we know) the only line that exhibits this particular circadian phenotype, we were unable to pinpoint a likely causal variant(s) due to a lack of statistical association power. In contrast, swapping chromosome 3 caused an almost complete phenotype recapitulation (Fig. 8A), implying that the major causal variant(s) is (are) most likely located on this chromosome. *cry* is located on this chromosome, which prompted us to reevaluate the identified 6-bp deletion in this gene as the possible causal mutation for the observed DGRP-796 circadian phenotypes. This mutation removes the Met^421^ and Trp^422^ residues (M421_W422del) and changes the Val^423^ to an Ile (V423I). In addition, we detected a nonsynonymous SNP (T > C) 8 bp downstream of the deletion, which transforms Ser^424^ to a Pro (S424P) (Fig. 8B and fig. S3D). The importance of these residues for Cry function is unclear as they are located between the chain of conserved tryptophan (Trp) residues, which mediates the photoinduced electron transfer activation, and the C-terminal lid domain that changes its conformation to bind Tim (*50*). To assess the impact of these amino acid alterations on Cry activity, we generated an atomic three-dimensional model of the DGRP-796 Cry protein by homology modeling based on the published Cry wild-type (WT) structure [Protein Data Bank (PDB) code: 4JZY] (*51*) and used molecular dynamics simulations to identify potential conformational differences between the WT and DGRP-796 Cry proteins. Intriguingly, we found that, although the global structure and dynamic behavior of WT and DGRP-796 Cry proteins are almost identical [root mean square deviation (RMSD) = 1.9 ± 0.1 Å; Fig. 8C and fig. S3E], the DGRP-796 *cry* mutation locally disrupts the secondary structure of the Asp^410^-Arg^430^ ⍺ helix. Our analyses revealed that it causes a marked reorientation of Trp^420^, which increases the putative distance between Trp^420^ and the FAD molecule and disrupts the alignment with the adjacent Trp residues (Fig. 8D). This likely impedes the photoactivatable electron transfer chain mechanism necessary for Cry photoactivation (*52*), suggesting that the *cry* mutation found in DGRP-796, which was initially perceived as being benign, might nevertheless generate a loss-of-function Cry. Moreover, we used molecular docking to evaluate whether FAD binding on the Cry pocket was affected by these mutations. While we were able to recapitulate the crystallographic binding mode of FAD for Cry WT (RMSD = 0.7 Å; fig. S3F), FAD could not be accommodated in the same binding conformation in the DGRP-796 Cry pocket (RMSD = 7.5 Å; fig. S3G). Collectively, these modeling and simulation results suggest that the 6-bp deletion found in the DGRP-796 *cry* allele impairs both the ability to productively bind FAD and the optimal conformation required for the photoactivatable electron transfer cascade mediated by Trp residues.

Last, we aimed to provide genetic support to our molecular, physiological, behavioral, and modeling Cry-related findings. To do so, we first assessed whether the DGRP-796 *cry* mutation generated a loss-of-function phenotype in vivo by testing the locomotor activity rhythms of DGRP-796 under constant light (LL). While WT flies were arrhythmic in LL, as expected, DGRP-796 flies showed persistent rhythms of locomotor activity in LL comparable to the *cry^02^* null-mutant flies (Fig. 8E) (*53*). We then generated a genetic cross between *cry^02^* and DGRP-796 lines, which was also robustly rhythmic in LL, providing further support for the 6-bp deletion in *cry* to be the causal mutation (Fig. 8E, fig. S3H). Last, overexpression of WT *cry* in *tim*-expressing cells restored the WT arrhythmicity in *cry^02^* and DGRP-796 flies in LL at comparable levels (Fig. 8E, fig. S3H). Together, these results confirm that the *cry* mutation in DGRP-796 flies is responsible for the disrupted molecular oscillator in DGRP-796 and provide in vivo evidence for the importance of the Trp-mediated flavin photoreduction mechanism for the circadian clock.

## DISCUSSION

The circadian clock is a ubiquitous system of temporal control over cellular physiology that is implemented through coordinated regulatory feedback loops that operate across all tissues (*2*, *3*). Yet, while the core molecular components of the circadian clock are conserved across tissues, other cycling transcripts vary significantly across systems. How these tissue-specific expression rhythms are established and how they vary between individuals and as such affect circadian behavior is still poorly understood. In this study, we explored TSC regulation at both the genomic and transcriptomic levels in the *D. melanogaster w^1118^* line as well as across 141 genetically diverse (DGRP) lines at high temporal resolution. This resulted in a unique circadian gene expression catalog composed of 233 samples stratified over four tissue (gut, brain, Malpighian tubules, and fat body)–specific time series for *w^1118^* and 451 static transcriptomes of three tissues for the DGRP lines.

### A comprehensive atlas of tissue-resolved circadian gene expression in *D. melanogaster*

Despite *D. melanogaster*’s crucial role in elucidating circadian biology, a comprehensive atlas of tissue-resolved, genome-wide circadian gene expression has, to our knowledge, never been generated. Using a novel, affordable transcriptomic profiling method, BRB-seq (*21*), which allowed us to incorporate several biological replicates in our dataset at high temporal resolution, we identified 1757 cycling genes. Given the nature of our experimental design, involving 1 day of observation in LD followed by 1 day in DD, it is possible that the computed set of cycling genes still contains light-induced genes whose cycling properties propagated into the second observation day. For example, C*G5455*, *Inos*, *CG31038*, and *CG17386* have previously been reported as light induced (*54*) and were found cycling in our dataset. Yet, we estimate the proportion of solely light-driven genes in our dataset to be rather small due to our joint analysis of LD and DD. Only 14 of 1757 genes, of which seven were known core circadian regulators, cycled in all four tissues. The molecular function of the other seven genes remains to be elucidated, but their impact on various circadian parameters upon their knockdown suggests important roles in circadian biology, either as propagators of time information or possibly even as core circadian regulators. The latter is especially compelling for *Usp1*, since knockdown of this gene affected the percentage of rhythmic flies the most. *Usp1* (*Ubiquitin-specific protease 1*) is predicted to be involved in the dynamics of protein ubiquitination, which has been shown to affect circadian fluctuation of hundreds of proteins in flies (*55*).

In contrast to these 14 genes, we found that most (80%) identified cycling genes do so in only one of the examined tissues, despite the fact that the majority are not differentially expressed across tissues. The abundance of genes that cycle solely in one tissue and their prevalence as a group over genes that cycle in multiple tissues suggest a high degree of autonomy for an organ’s circadian rhythm. This observation is in line with previous studies that revealed that peripheral circadian clocks are, to a large degree, independent of the central clock, although this varies among tissues (*56*). For example, *Drosophila* Malpighian tubules featured a self-sustained, light-entrained clock that remained functional even upon transplantation into flies that were reared in antiphase LD conditions (*57*). In addition, the circadian clock in the fat body remained operational in the absence of input from the brain in a LD regime. However, in the absence of light cues, the brain circadian clock was required (*58*, *59*). For the *Drosophila* gut, a detailed tissue-specific transcriptomic analysis of circadian gene expression has, to our knowledge, not yet been performed (*56*) nor has a link to the central clock in the brain been thoroughly investigated. Nevertheless, the circadian clock was found to be present in all gut cell types, except enteroendocrinocytes, and could be synchronized by photoperiod and environmental input, such as feeding, while it was also shown to be essential for gut regeneration (*60*, *61*).

Although these autonomous circadian rhythms are clearly remarkable, its molecular basis is still poorly understood. We believe that our study provides, in this regard, previously unknown molecular insights into this phenomenon. Specifically, our GRN analyses based on the set of cycling genes that we identified showed that core circadian regulators, such as *vri*, *Clk*, *Pdp1*, and *cwo*, are, as expected, tightly interconnected and form the foundation of circadian gene expression in all tissues. However, we also observed that this central module, formed by the core circadian regulators, is connected to smaller hubs of tissue-specific cycling TFs. These hubs may be the defining elements of the circadian clock’s tissue specificity and serve as transmission units of the central circadian input throughout the entire circadian GRN. Consequently, the mechanism underlying circadian tissue specificity would be, in essence, an interaction between core circadian regulators (*Clk*, *Pdp1*, *cwo*, etc.) and tissue-specific TFs, e.g., *emc* in case of the brain. The mode of interaction between these core and tissue-specific regulators could follow at least two scenarios: (i) cobinding, as previously shown for Opa/Srp establishing body-specific circadian rhythms (*2*), whereby tissue-specific TFs could involve both cycling and noncycling TFs, as suggested by our motif enrichment analysis; and (ii) a core circadian regulator initiating a regulatory cascade by activating the expression of tissue-specific TFs, which then propagate the signal.

TFs forming individual tissue hubs did not have a common biological function based on GO term analysis. Nonetheless, four of the fat body–specific cycling TFs (*svp*, *noc*, *salm*, and *Kr-h1*), the primary genes of the module, are zinc finger TFs that are involved in the compound eye photoreceptor differentiation process (*62*). The participation of zinc finger proteins in circadian clock modulation has been shown before in both *Drosophila* (*63*) and mouse (*64*). Therefore, we hypothesize that those genes have a secondary function of mediating light entrainment in the fat body independent from the central molecular clock. With respect to the gut, we observed that an uncharacterized TF, *CG9932*, has almost as many outgoing edges to gut-cycling genes as the well-known circadian regulator *cwo*. *CG9932*’s high connectivity suggests that it too may play an important role in the intestinal circadian clock. These are just a couple of examples of how our comprehensive, tissue-resolved atlas can be used to decipher not only tissue-specific gene regulatory programs responsible for complex circadian behavior and physiological patterns in *Drosophila* but also other species.

Considering the high degree of autonomy of the molecular circadian clock, we wondered whether this independence extended to genes that cycled across several tissues. However, we found that about a third (29%) of the genes that cycled in several tissues are asynchronous. This is clearly an unexpectedly high fraction, although it is possible that the inbred and laboratory-adapted nature of DGRP flies induces stronger phenotypes than what would likely be observed in WT lines, as has been documented in previous studies using DGRP lines (*65*). Asynchronously cycling genes covered various biological processes, yet one of them, glutathione metabolism, was enriched with implicated genes displaying a notable (>4 hours) expression lag in the fat body compared to the gut and Malpighian tubules. The biosynthesis of glutathione, a prime antioxidant and controller of signaling and cellular detoxification (*66*), has previously been reported to be under clock control in fly heads (*30*, *31*, *67*), although information about the tissue specificity of this process was lacking. We hypothesize that this phase control is primarily achieved through circadian transcriptional regulation of catalytic (*Gclc*) and modulatory (*Gclm*) subunits of the Gcl holoenzyme, as well as glutathione *S*-transferase D1 (*GstD1*) given that these genes are directly targeted by Clk/Cyc (*2*). Further studies will be required to elucidate the underlying molecular mechanisms in more detail as well as their physiological relevance.

### Large-scale genetic variation affects circadian rhythm

One of the main challenges in the field of circadian biology research is the need for (i) long-term observations that exceed two periods of oscillations (i.e., 48 hours and longer) and (ii) relatively frequent sampling to acquire regulatory insights. These requirements become especially pertinent in studies aimed at exploring how genetic variation affects the molecular circadian clock. In this study, we addressed these challenges through the implementation of a nonclassical experimental design involving static but high temporal frequency probing of available genotypes (*40*) and the use of our high-throughput, transcriptomic approach, BRB-seq (*21*). Applying this strategy on the DGRP allowed us to effectively infer the physiological time of 141 sampled fly lines across several tissues. One of the most unexpected results that stemmed from this analysis is that TSC rhythms are highly variable in the DGRP population. Thirty-two percent (45) of the lines showed aberrant circadian gene expression with about half exhibiting a predicted physiological time shift larger than 3.4 hours. Given the technical limitations of our approach, we believe that this may even be a conservative estimate. However, to which extent this time shift has a physiological or behavioral phenotypic impact is unclear at this point, especially since the observed molecular circadian variation manifested itself mostly in tissue-specific fashion. Most of the identified line outliers featured an altered molecular clock in only one or two tissues. While the autonomy of the peripheral clocks in *Drosophila* has been reported (*56*) and discussed before, such natural asynchrony (in contrast to imposed asynchrony) (*57*) in molecular circadian rhythms between various tissues has, to our knowledge, not been observed before. The gut thereby stood out as the organ featuring the highest number of lines (26) with an altered physiological time. We hypothesize that this is caused by the interaction between genetic components of the circadian clock and strong endogenous cues such as feeding behavior and the microbiota (*67*, *68*).

While our static sampling approach offered several experimental and analytical advantages, it also suffers from a number of drawbacks. First, our capacity to infer physiological time depends, in large part, on the obtained signal-to-noise ratio in each tissue. This ratio may be the lowest in the brain given that only a minority of neurons express circadian genes (*69*), and moreover, different neuronal groups are phase shifted from each other, like morning and evening cells (*70*, *71*). In addition, there is evidence from mouse that glia cells may be cycling in different phases (*72*). These findings likely clarify, to some extent, why only few lines exhibited a detectable time shift in the brain despite featuring an altered, behavioral circadian period such as DGRP-774. This interpretation is further supported by the detection of fewer cycling genes in the brain in the *w*^1118^ time series compared to other tissues. Second, the detection of cycling genes and inference of their period is not possible based on one time point. Third, single static transcriptomes do not allow distinction between different changes in the molecular clock. Consequently, phase shifts in the molecular clock relative to the reference rhythm, alterations in period length, or even full obliteration of the clock will all appear in our analysis as deviations from the expected, as observed for lines DGRP-774 and DGRP-796 with the latter having even an abolished circadian clock. Last, the capacity to mark a sample as diverging from the expected reference time line is stochastic and depends on the sampling time. For instance, a sample with a phase-shifted circadian clock can still take the same value as the reference at a certain time of the day. If sampling was performed during that specific time interval, the deviating sample will be indistinguishable from the reference (fig. S2C). Our data on DGRP-774 (Fig. 5D) illustrate this point given that the ZT9, ZT11, ZT13, and ZT19 samples were all identified by our model as outliers; whereas the other time points, including ZT1, ZT3, ZT7, ZT15, ZT21, and ZT23 were not. Our readout is thus prone to false negatives and binary, as our model simply allows us to flag significantly deviating samples, while the retrieved time shift values should only be interpreted as indicative. Despite these limitations, we still believe that our findings yield a strong foundation for further research aimed at dissecting circadian clock genetics as well as the regulatory principles of the observed molecular asynchrony and its influence on physiology.

To resolve possible molecular mechanisms underlying the observed circadian gene expression variation, we focused on DGPR-796, revealing a novel *cry* allele *cry*^DGRP-796^ that manifests as a loss-of-function phenotype based on genetic, molecular, physiological, and behavioral analyses. In *Drosophila*, Cryptochrome is the principal blue light sensor and directly transmits light signals to the molecular clock (*73*). Light transduction is initiated via oxidation of photoexcited FAD cofactor bound by Cry. Subsequently, FAD is reduced by the first tryptophan (W420) in the Trp tetrade cascade resulting in further electron propagation through the tryptophan tetrade (*52*, *74*). We found that the discovered *cry* variant in DGRP-796 is a 6-bp in-frame deletion, which gives rise to a composite change of the amino acid residues at positions 421 to 423 of the protein. This lastly results in the deletion of M421 and W422 adjacent to the W420 (M421-W422del) and a V423I substitution. The difference in amino acid sequence results in a local disruption of the ⍺ helix and, most importantly, markedly reorients W420. Because of the complex nature of the allele, implicating at once three amino acid changes, it is difficult to determine the exact influence of each of the implicated amino acid changes on protein function. We argue though that the phenotypic effect is mainly caused by the W422 residue as it is conserved across multiple animal species, while M421 and V423 are not (*74*). Although W422 is located in the same FAD pocket as W420 in the WT Cry, it is unlikely that W422 participates in the electron transport chain due to its relative location to the FAD molecule. In contrast, our simulations showed that the allele might affect FAD binding efficiency to Cry representing the first instance of a genetic change in *Drosophila* affecting FAD-Cry interactions. The detection of this *cry*^DGRP-796^ allele shows, in our opinion, the potential power of our generated dataset and strategy to reveal unknown genetic determinants of circadian phenotypic variation. Moreover, it provides, to our knowledge, the first in vivo evidence via immunohistochemistry and locomotor activity assays for the importance of the Trp-mediated flavin photoreduction cascade in Cry photoactivation.

Nonetheless, we note that the behavior of DGRP-796 does not completely match that of the *cry* single mutant given that the evening locomotor activity peak does not end sharply at the “lights off” time but rather gradually (Fig. 6A) (*49*, *75*, *76*). Such behavior was previously observed in mutants featuring a disruption of genes that are implicated in light perception pathways such as *eya*, *so*, *Hdc*, and *gl* (*75*). Given that swapping the *w^1118^* chromosome 2 with that of DGRP-796 resulted in a partially reduced phase response to the light pulse (Fig. 8A), it is thus possible that the *cry* variant in DGRP-796 is accompanied with an auxiliary phenotype-inducing variant that is also located on chromosome 2. Our search of disruptive chromosome 2 variants in light- or circadian rhythm–associated genes revealed 44 genes harboring various types of protein coding–affecting variants. However, the lack of statistical power precluded us from identifying causal genetic variant(s) contributing to this particular circadian phenotype.

Together, this study substantially expands the catalog of known circadian transcripts and adds new layers of information to circadian biology including tissue specificity, natural occurring phase shifts, and the influence of genetic variation. Moreover, the generated atlas should constitute a rich resource for extrapolating the acquired knowledge to other systems, including mouse and human.

## MATERIALS AND METHODS

### Experimental design

#### Drosophila *stocks and general experimental conditions*

*w^1118^* (*W*^−^) and UAS-RNAi flies were obtained from the Transgenic RNAi Project and Bloomington Stock Center, while the GD and KK UAS-RNAi flies were ordered from the Vienna *Drosophila* Resource Center. The complete list of genotypes is presented in table S3.

The supplementary details of each line can be found on the websites of their corresponding stock centers by stock identifiers listed in table S3. Stock identifications for the DGRP lines used in this study are available in table S1. The *tim*-Gal4 driver provides a means to direct RNAi action to *tim*-expressing cells across the whole body, including all brain cells, while *Pdf*-Gal4 targets LN_v_s.

*Drosophila* flies were raised on food containing 58.8 g of yeast 96 (Springaline BA10), 58.8 g of Farigel wheat (Westhove FMZH1), 6.2 g of agar powder (ACROS 400400050), 100 ml of grape juice (Ramseier), 4.9 ml of propionic acid (Sigma-Aldrich P1386), and 26.5 ml of methyl 4-98 hydroxybenzoate (VWR ALFAA14289.0; stock: 400 g/liter in 95% ethanol), dissolved in 1 liter of water. The temperature was set to 25°C, and light exposure was set to 12-hour/12-hour LD cycles unless stated otherwise.

#### Experimental design and tissue dissection: General notes

Two time course experiments were performed in our study. The first time course involved only the *w^1118^* genotype, and it allowed us to evaluate tissue specificity of the circadian transcriptome (see “Experimental design: Time course experiment of *w^1118^*” section for a detailed description below). The second time course experiment involved 141 DGRP lines and was aimed at studying the impact of genetic variation on molecular circadian rhythms at a tissue-specific level (see “Experimental design: Time course experiment of DGRP lines” section for a detailed description below).

For both of the time course experiments, the incubators were placed behind light-blocking curtains to minimize experimental disruptions when samples were collected in DD conditions. For all of the samples mentioned above, unless stated otherwise, we dissected the brain, abdominal fat body, whole gut without the crop, and Malpighian tubes. Within each genotype and time point, the same flies were used for collecting all organ and tissue samples, see table S1 for the exact number of the dissected flies per sample. Thus, for any given fly line, the *N* brains were derived from the same individuals as the source of *N* guts, *N* fat bodies, and *N* Malpighian tubules. The gut in this experiment consists of the foregut and midgut, starting at the proventriculus and finishing at the midgut-hindgut junction. To harvest the abdominal fat body, we first removed the gut and sexual organs from the abdomen and then separated the fat body from the dorsal part of the abdominal cavity using pincers.

#### Experimental design: Time course experiment of w^1118^

We used the reference *w^1118^* fly line to study the extent of tissue specificity of molecular circadian rhythms. Two 3-day-old mated males were separated from females under CO_2_-induced anesthesia and placed into vials grouping up to 20 flies per vial. Vials were placed in an incubator with a 12-hour/12-hour LD cycle at 25°C. On day 3, at ZT0 (Zeitgeber Time, 0 indicates the beginning of the measurement), these vials were divided into two groups. One set of vials was kept at LD settings for 24 hours, and the remainder was placed in an incubator at 12-hour/12-hour DD settings. Samples derived from the group placed in LD condition received the ZT code, and samples placed in the DD condition received the CT code. One hour after the start of the experiment (10:00 a.m.), one vial from each group was collected by transferring the flies into a 15-ml tube and by flash-freezing these in liquid nitrogen. This collection point is equivalent to time points ZT1 and CT1 for the LD and DD conditions, respectively. Three hours after the start of the experiment, at 12:00 p.m. (ZT3/CT3), the next collection point occurred, and one vial per group was collected, transferred into a 15-ml tube, and flash-frozen in liquid nitrogen. This continued over the course of 24 hours until no vials with flies were left. Samples that were kept in the dark conditions were also collected in a dark environment. To perform subsequent RNA-seq, flies from each sample were divided into three biological replicates per tissue and per time point (table S1).

#### Experimental design: Time course experiment of DGRP lines

Our experimental design for the time course using DGRP lines was based on one used to map gene expression changes to genetic variation in *Caenorhabditis elegans* by Francesconi and Lehner (*40*). Doing so for circadian rhythms, this meant that each DGRP line that was available to us would be sampled only once in a 24-hour period, effectively resulting in the sampling of a random DGRP line every 9.5 min. For each DGRP line, two 3-day-old mated males were separated from females under CO_2_-induced anesthesia and placed into vials grouping up to 20, but no less than 10 flies per vial. To allow the flies to stabilize, vials were placed in an incubator at 12-hour/12-hour LD settings.

In parallel, we randomly selected four genotypes to act as an internal control. On the basis of the analysis from Harbison *et al*. (*16*), three of these DGRP lines (DGRP-208, DGRP-321, and DGRP-536) exhibited regular circadian rhythms, were kept in our analysis, and were collected every 2 hours over 24 hours starting from ZT1 with the exception of DGRP-208 that was collected every 4 hours and in DD settings. Sample collection was done in a similar fashion as described above for the *w^1118^* dataset. For both the DGRP group and the internal control group, samples were processed only with a single replicate for each tissue per genotype per time point.

#### Experimental design: Time course experiment of DGRP-796

DGRP-796 was processed similarly to the *w^1118^* time series experiment but mostly in duplicates. Sample collection was performed as described above.

### RNA samples and libraries preparation

Around 10 male flies from each genotype were collected at defined time points (table S1) and flash-frozen at −80°C before processing. Brains, guts, fat bodies, and Malpighian tubules were dissected on ice in 1× phosphate-buffered saline with 0.02% Tween 20, immediately transferred into screw cap tubes with glass beads and 350 μl of TRI Reagent (Molecular Research Center, TR118), and placed in a Precellys 24 for homogenization (settings: 6000 rpm/30 s). Homogenized tissues were flash-frozen at −80°C until subsequent RNA purification. To avoid batch effects due to differences in RNA purification efficiencies, all homogenized samples were purified in parallel using the Direct-zol 96 RNA Purification system (Zymo Research, R2056) according to manufacturer’s instructions. Total RNA was eluted in water and quantified with the Quant-iT RiboGreen RNA Assay Kit (Thermo Fisher Scientific, R11490). Again, to avoid batch effects in cDNA amplification and library preparation, we took between 10 and 50 ng of the total RNA from each sample and used the high-parallel and high-throughput BRB-seq library preparation method as described (*21*).

### RNA-seq data preprocessing

Raw sequencing reads for all 778 samples (table S1) were obtained from the Illumina HiSeq 2500 (paired-end; 75 cycles). The samples were pooled into 16 libraries and distributed across the sequencing lanes to prevent a lane-induced batch effect. Multiple fastq files for each library were merged by SAMtools v1.3 after which they were subjected to demultiplexing using BRB-seq tools v1.1 based on the information contained within the first read in the pair (R1) (https://github.com/DeplanckeLab/BRB-seqTools) (*21*). Quality control of every sample was performed using FastQC v0.11.2.

### Immunohistochemistry and confocal microscopy

Whole flies were fixed in 4% paraformaldehyde for 2 hours at room temperature at ZT21 after a 30-min light pulse followed by a 1-hour return into DD as previously described in (*48*). Whole brains were dissected and blocked in 5% goat serum overnight at 4°C (G9023, Sigma-Aldrich). To exclude possible cross-talk between the antibodies and bleeding from the Pdf staining, experiments with two sets of secondary antibodies were performed. For the stainings presented on Fig. 7 (A and B), brains were then incubated for 2 days at 4°C with 1:200 polyclonal rat anti-Tim antibody donated by M. Rosbash (*48*) and 1:1000 monoclonal mouse anti–Pdf-C7 (Development Studies Hybridoma Bank, University of Iowa). Secondary antibodies conjugated with Alexa Fluor 488 (for Pdf) or Alexa Fluor 647 (for Tim) were used at a 1:200 dilution. For the stainings presented on fig. S3B, a different pair of antibodies was used: Alexa Fluor 488 for Pdf and Cy3 for Tim, respectively, while the rest of the experimental conditions stayed the same. For Tim-only stainings, Cy3 secondary antibodies were used.

Images were processed using ImageJ (https://imagej.nih.gov/ij/). First, *z* planes containing neurons of interest were selected. Next, custom lookup tables were assigned to both channels. The minimum and the maximum of the Tim channel were then set to 0 and 30, respectively. Similarly, the minimum and the maximum of the Pdf channel were set to 0 and 50, respectively. Last, the “standard deviation” Z-projection method was applied to each of the channels. A full list of commands used to process the images is available as an ImageJ plugin on the GitHub. Tim- and Pdf-stained neurons in l-LN_v_s and LN_d_s were counted manually.

### Statistical analysis

For all scripts written in R, we used version v3.4.1 unless otherwise noted.

### Mapping to the reference genome and genotyping

We ran all the sequenced samples through a computational genotyping pipeline to ensure the accuracy of the sample genotypes. This pipeline assesses the previously available variants detected in the DGRP lines and compares this with the ones detected in each sample. To be able to achieve the highest reliability for the variant calling procedure, we followed the Genome Analysis Toolkit (GATK) best practices workflow for SNP and indel calling on RNA-seq data. The alignment was executed in two stages:

• First, the *D. melanogaster* reference genome [dm3, University of California Santa Cruz (UCSC), Berkeley Drosophila Genome Project (BDGP) Release 5 Apr 2006] was indexed by STAR v.2.5.0b (*77*) using the RefSeq Genes annotation track (UCSC, annotation database for dm3). The samples were aligned to the genome by STAR in the alignReads mode as single end data with the following parameters: *--outFilterScoreMinOverLread 0.20, --outFilterMatchNminOverLread 0.20* and *--outFilterMultimapNmax 1*. Next, all detected splice junctions from the samples were merged into one file and used to create a second index for the reference genome by STAR in genomeGenerate mode with the maximum possible overhang of the reads set to 75 bp (*--sjdbOverhang 75*).

• Second, reads were mapped by STAR using the same parameters as above to the reference genome obtained at the end of the previous step. Then, SAMtools was used to remove reads with an insert size >1 kb. Next, soft clipping beyond the end of reference alignment and setting mapping quality (MAPQ) to 0 for unmapped reads was performed with Picard v2.2.1 CleanSam (http://broadinstitute.github.io/picard). After that, read group information was added (Picard AddOrReplaceReadGroups), and duplicates were marked (Picard MarkDuplicates) using default settings, followed by “mapping qualities reassignment” by GATK v3.6-0 (SplitNCigarReads *-rf ReassignOneMappingQuality -RMQF 255 -RMQT 60 -U ALLOW_N_CIGAR_READS*). Last, local realignment around indels was performed with GATK RealignerTargetCreator and IndelRealigner using default parameters.

After these two stages, GATK HaplotypeCaller was used on every sample in genomic variant call format (GVCF) mode to call variants with the minimum phred-scaled confidence threshold set at 30 and the emission confidence threshold set at 10. Indels, multiple nucleotide polymorphisms (MNPs), and variants with a depth of coverage less than 5 were excluded from further consideration. Afterward, GATK CombineGVCFs was used to produce a multisample GVCF. Last, GATK GenotypeGVCFs with the same phred-scaled confidence threshold and emission confidence threshold as above were applied to obtain a multisample set of variants. Only biallelic SNPs with a depth of coverage > 5, a Fisher strand score of >30.0, and a quality by depth <2.0 were selected from the set to compare to the reference DGRP2 VCF (*14*, *15*).

Furthermore, we compared each of our samples to all of the available DGRP lines from the reference DGRP2 VCF (*14*, *15*). Then, we assessed the top three of genotype matches. If the tested DGRP and the expected DGRP were the highest ranked, had a >90% match, and the second and third match were at least 5% lower, we considered it as a clean match. In case the first ranked expected DGRP did not match the tested DGRP, however, and >90% of the tested loci matched the expected DGRP, and the second and third matches were 5% lower, then we considered this to be a mislabeling artefact and renamed the DGRP accordingly.

### Read counting, normalization, and batch correction

The bam files obtained at the second step of the genotyping procedure after “read with an insert size of >1-kb removal” were used to count the number of reads falling into each of the 16,995 genes using the Python package HTSeq v0.6.1 ran under union mode (*78*). In general, the number of raw reads per sample ranged from 445,538 to 12,709,229 with a mean of 1,978,519. Samples with <300,000 reads assigned to the genes (no feature, ambiguous, low quality, not aligned, or “mapped to multiple locations” reads were not considered) were excluded from the analysis, resulting in numbers of uniquely mapped to genes reads ranging from 370,312 to 10,567,493, with a mean of 1,488, 250 across the dataset as the mapping efficiency varied from 48 to 91% with a mean of 74.57%. This threshold resulted in the following: (i) 233 samples for the *w^1118^* dataset, with 63 samples dedicated to the brain, 59 to the fat body, 58 to the gut, and 53 to the Malpighian tubules, respectively; (ii) 18 samples for the DGRP-208 time course with 6 samples per tissue: brain, gut, and fat body; (iii) 35 samples for the DGRP-321 time course with 12 samples dedicated to the brain and fat body each and 11 samples dedicated to the gut; (iv) 31 samples for the DGRP-563 time course with 12 samples of the brain, 11 of the fat body, and 8 of the gut, respectively; (v) 29 samples for the DGRP-774 time course with 11 samples for the brain and fat body each and 7 samples for the gut; (v) 94 samples for the DGRP-796 time course of which 46 samples were designated to the brain and 48 to the gut; and (vi) 338 static transcriptomes of the DGRP with 102, 125, and 100 samples for the brain, fat body, and gut, respectively, comprising 778 samples used for the downstream analysis.

To assess the breadth of our dataset in terms of gene coverage as another metric of quality control, we counted how many genes in the dataset have >1 read assigned to them. Across all tissues and samples, the number of expressed genes ranged from 6915 to 13,131, with a mean of 9933. In the brain, samples demonstrated from 7615 to 11,460 expressed genes, with a mean of 9835; in the fat body, the number of expressed genes per sample ranged from 6915 to 12,550, with a mean of 10,013; in the gut, this metric ranged from 7629 to 13,131, with a mean of 9809; and lastly, in the Malpighian tubules, the number of expressed genes per sample ranged from 7464 to 12,894, with a mean of 10,630.

To keep the samples comparable while not discarding lowly expressed genes, three count tables for each tissue were created. The first table contained only *w^1118^* reference time series samples; the second table contained *w^1118^* reference time series, time series for the four profiled DGRP lines, and single observation DGRP samples; and lastly, the third table contained time series data for *w^1118^* and DGRP-796 samples. All the tables were processed in the same way described below. Genes that were expressed in less than 80% of the samples were removed from the analysis. Then, the tables were quantile normalized with the voom function from the limma v3.32.5 package (*79*). Last, within each tissue, batch effects from different libraries were removed with ComBat from the SVA v3.24.4 package (*80*).

### Reference time series (*w^1118^*) and DGRP-796 RNA time series expression analysis for the detection of circadian genes

To detect genes with circadian expression patterns, JTK CYCLE v3.1 (*22*) was used separately for each tissue. Before that, expression values for each gene were scaled to a range from 0 to 1 across the samples in the time series. Samples from LD (first 24 hours of observation) and DD (second 24 hours of observation) conditions were analyzed as one set spanning 48 hours as an observation period of 48 hours is essential for the reliable detection of circadian cycling. Such experimental design also allows us to avoid the detection of light-induced genes as they would not be cycling in DD and therefore would not be labeled as circadian. A threshold of <0.05 on the permutation-based *P* values of the Jonckheere-Terpstra test (ADJ.P) implemented in the JTK CYCLE was applied. No threshold on the amplitude was applied.

### Phase shift analysis

CircaCompare v0.1.0 (*29*) was used to detect a phase shift in the expression pattern of genes cycling in two or more tissues. For genes cycling in three or four tissues, all possible pairs of tissues were considered. A phase shift was considered statistically significant if the difference in the peak times estimated by CircaCompare exceeded 2 hours with an FDR < 0.05. GO enrichment for the phase-shifted genes was performed with the R package topGO v.2.34.0. All genes cycling in several tissues were used as background.

### Circadian network construction based on time series gene expression

TSC GRNs were inferred with the dynamic GENIE3 algorithm based on genes that were detected as circadian in a tissue (*33*). The decay rate of gene expression was set to half the period estimated by the JTK CYCLE. The list of candidate TFs were derived from FlyBase, accession term FBgg0000745.

For network visualization with Cytoscape v.3.6.0 (*32*), only the top 200 links ranked by weight assigned by dynGENIE3 were used. The links were collected from all four tissues for the *w^1118^* time series data, while for the *w^1118^* and DGRP-796 merged networks, only links detected in gut samples were used. For visual clarity of the network, TFs were grouped by TSC patterns, followed by the Kamada-Kawai network layout algorithm application. In addition, a grid layout was applied to the groups of TFs. Node centrality and betweenness were calculated with the degree and betweenness functions from the igraph v.1.2.2 package.

### Physiological time assignment and method evaluation

To achieve optimal precision in the estimation of the physiological time for each DGRP sample based on gene expression profiles from the single observation made on DGRP lines, we evaluated five methods: MTT (*41*), ZeitZeiger (*42*), in-house–developed predictors based on LASSO and DirectStat, and lastly, neural networks.

#### Molecular time table

Because of the differences in our experimental setup, the implementation of the method was slightly modified from the original: In our study, two consecutive days of observations were performed on the reference time series (*w^1118^*, LD and DD) versus 4 days in the study published by Ueda *et al*. (*41*). To select time-indicating genes (TIGs), the time series expression profile of each gene in the training set was analyzed through two filters, one for circadian rhythmicity and the other for high amplitude. To detect circadian rhythmicity, the expression of each gene was correlated with the bundle of the artificial cosine waves with a 24-hour period created by 10-min phase increments (144 in total), and the maximum correlation value was recorded. The suggested cutoff of a correlation > 0.8 resulted into a small number of genes passing through and thus was reduced down to 0.5. High amplitudes of putative TIGs were determined by the coefficient of variation in the expression > 0.20. Once TIGs were identified, their molecular peak time (MPT) was recorded as the peak time of a best correlated cosine wave. After that, expression values of the TIGs in the test sample were correlated with the bundle of cosine waves with a 24-hour period and a phase shift of 10 min generated on MPTs. The phase shift of the most correlated cosine wave was then registered as the physiological time of a sample. Several sets of TIGs were subjected to evaluation: (i) TIGs obtained by applying the filters separately to LD and DD and afterward overlapped (LD_DD_05); (ii) TIGs passing the filters applied to LD and DD time series considered as one unity (LDDD_05); (iii) the top 25, 50, and 75% and all genes detected as circadian by the JTK CYCLE; (iv) TIGs listed in Supporting Table 3 of Ueda *et al.* (*41*) and expressed in our dataset; and (v) TIGs listed in Supporting Table 3 of Ueda *et al*. (*41*) and detected as circadian by JTK CYCLE. The method was implemented in R v.3.4.1 with base functions.

#### ZeitZeiger v.1.0.04 (*42*)

First, cross-validation on the training dataset was performed to determine the best parameters of the algorithm resulting into the optimal amount of regularization = 1.5 and optimal amount of the sparse principal components (SPCs) = 2. Then, the zeitzeigerFit function was used on the training dataset to fit a spline into the gene expression profiles of all expressed genes. Next, the SPCs were calculated using zeitzeigerSpc, and lastly, zeitzeigerPredict was used to predict physiological time of a test sample.

#### Neural networks

We predicted physiological time with the use of neural networks applied to the genes detected as circadian by the JTK CYCLE. Three neural network configurations were tested: (i) a single layer network with 12 input neurons, (ii) a network with 12 input neurons and 6 neurons in the hidden layer, and (iii) a network with 12 input neurons and 2 hidden layers having 6 and 3 neurons, respectively. The training of the networks was done by the neuralnet function from the neuralnet v1.3 package in R. The prediction of the physiological time of the test samples was then achieved via the compute function from neuralnet v1.3.

#### Least absolute shrinkage and selection operator

We also developed and evaluated the LASSO regression analysis for the prediction of the physiological time. It consisted of two parts: first, the period of the day at which the sample was harvested (a.m. or p.m.) was determined to account for the symmetry of the circadian gene expression patterns. In the second step, LASSO calculated the physiological time based on the result of the first step. The analysis was implemented with glmnet v.2.0-13 used on the count table containing the time series data of all expressed genes.

#### Directional statistics

A cyclic nature of time concept served as inspiration to the development of the DirectStat approach for the prediction of physiological time. In this method, we considered time of the day as a point in polar coordinates and used DirectStat to build a regression. First, time of the day was converted to radians where 24 hours corresponds to 2π rad. Then, we used forward backward early dropping selection for circular data as implemented in the function spml.fbed from the R package Directional v4.1 built under R v.3.5 to select genes that could be predictors of time. Last, we fit the regression with the function spml.reg and predicted physiological time using the obtained model.

A cross-validation leave-one-out approach was used to benchmark the methods listed above. The evaluation set was composed of the reference *w^1118^* time series complemented with the samples for three DGRP lines that were profiled every 2 hours over a period of 24 hours during the overall DGRP single time point collection (DGRP-208, DGRP-321, and DGRP-563) together totaling 317 samples across four tissues. Gene expression values in the set were standardized to the range of 0 to 1 before the application of the methods. During the procedure, one sample was removed from the set (test sample), and training of the models was performed on the rest of the samples (training set), followed by the prediction of the physiological time on the test sample. Then, the difference in time between the estimated physiological time and time of harvesting was calculated. Thus, every method was scored 317 times. Last, the best approach was determined as the one with the least difference between the predicted and expected physiological time across all tissues (estimated based on the mean and SD; see fig. S2B), which, in our case, was the MTT method based on TIGs passing the filters applied to LD and DD time series considered as one unit (LDDD_05). To evaluate even further the performance of the method, we assessed predictions of the model for the three profiled DGRP line samples: the mean difference between predicted physiological time and recorded time of harvesting was <1.4 hours across all tissues. Predictions for each tissue were conducted separately. A DGRP sample was conservatively marked as an outlier if the predicted physiological time and the time of harvesting differed more than 3.4 hours. This cutoff was dictated by the sensitivity of the MTT and represents three SDs of the distribution of differences between the predicted time and time of harvesting across all four tissues, *w^1118^*, and three profiled DGRP lines samples.

As a proof of concept demonstrating the ability of the MTT method to detect deviations in molecular circadian rhythms based on static transcriptomes, we applied it to the time line of DGRP-774 with respective samples taken every 2 hours for 24 hours across three tissues (11 samples for the brain, 11 for the fat body, and 7 for the gut, respectively). DGRP-774 is known to have an 18.86-hour period in males (*16*) and is therefore expected to have an altered molecular circadian rhythm. Each sample was considered as an individual static transcriptome, and MTT was applied. If the difference between the estimated physiological time and the time of harvesting for a sample exceeded 3.4 hours, it was marked as an outlier. As a result, one fat body and three gut samples were detected as outliers supporting the notion that MTT can identify deviations in molecular circadian rhythms based on static transcriptomes.

We must note that the efficiency of detecting molecular circadian rhythm differences between a fly line with circadian rhythm deviations, such as an extended or shortened period or a phase shift, from static transcriptomes does not clearly correlate with the absolute value of the deviation (fig. S2C). To illustrate this, we simulated a circadian gene expression value as a sinus wave with an amplitude of 100 U every 10 min for 24 hours for three datasets: one featuring “reference” expression rhythms with a period of 24 hours, one displaying a shortened period of 20 hours and one an extended period of 30 hours (fig. S2C). The amplitude of 100 U was chosen for clarity. Also, our basic model included noise, where the amplitude-to-noise ratio was derived as the mean across time and genes “amplitude-to-noise” ratio of the seven core clock genes (*tim*, *vri*, *Clk*, *Pdp1*, *cry*, *cwo*, and *per*) that were assessed in the *w^1118^* dataset. Panels one and three of fig. S2C illustrate that, despite the fact that the period in panel 3 is 30 hours (6-hour deviation), the time window during which we could detect a difference between the static transcriptome of the deviating line and a reference transcriptome is only 7 hours. This is comparable with the detection time window for rhythms with a 20-hour period (4-hour deviation). Moreover, the addition of a 6-hour phase shift into the model led to an increase of the detection window to 10 hours for the 20-hour rhythms and no change in the detection window size for the 30-hour rhythms, as shown in the second and fourth panels (fig. S2C), respectively. This simulation, however, is just an illustration of possible situations and their dependence on unknown before the experiment start variables, such as the molecular period and phase. It thus did not aid in choosing which DGRP lines to use to create a proof-of-concept expression time line dataset.

## SUPPLEMENTARY MATERIALS

Supplementary material for this article is available at http://advances.sciencemag.org/cgi/content/full/7/5/eabc3781/DC1

## Appendix

View/request a protocol for this paper from *Bio-protocol*.
